# Designing a global monitoring system for pilot introduction of a new contraceptive technology, subcutaneous DMPA (DMPA-SC)

**DOI:** 10.1016/j.evalprogplan.2018.03.004

**Published:** 2018-06

**Authors:** Anna Stout, Siri Wood, Allen Namagembe, Alain Kaboré, Daouda Siddo, Ida Ndione

**Affiliations:** a PATH, PO Box 900922, Seattle, WA 98109, USA; b PATH, PO Box 7404, Kampala, Uganda; c UNFPA Burkina Faso, Rue Maurice Bishop, Immeuble des Nations Unies, 01BP 575 Ouagadougou 01, Burkina Faso. Currently with PATH, Dakar, Senegal; d UNFPA Niger, Maison des Nations Unies, 428 Avenue du Fleuve Niger, B.P. 11207 Niamey, Niger; e PATH, BP 15115, Dakar-Fann, Dakar, Senegal

**Keywords:** Family planning, Injectable contraception, Contraceptive introduction, Product introduction, Pilot, Monitoring systems, Health information systems (HIS), Indicators, Sayana Press, Subcutaneous DMPA, DMPA-SC, Burkina Faso, Niger, Senegal, Uganda, Africa

## Abstract

In collaboration with ministries of health, PATH and key partners launched the first pilot introductions of subcutaneous depot medroxyprogesterone acetate (DMPA-SC, brand name Sayana^®^ Press) in Burkina Faso, Niger, Senegal, and Uganda from July 2014 through June 2016. While each country implemented a unique introduction strategy, all agreed to track a set of uniform indicators to chart the effect of introducing this new method across settings. Existing national health information systems (HIS) were unable to track new methods or delivery channels introduced for a pilot, thus were not a feasible source for project data. We successfully monitored the four-country pilot introductions by implementing a four-phase approach: 1) developing and defining global indicators, 2) integrating indicators into existing country data collection tools, 3) facilitating consistent reporting and data management, and 4) analyzing and interpreting data and sharing results. Project partners leveraged existing family planning registers to the extent possible, and introduced new or modified data collection and reporting tools to generate project-specific data where necessary. We routinely shared monitoring results with global and national stakeholders, informing decisions about future investments in the product and scale up of DMPA-SC nationwide. Our process and lessons learned may provide insights for countries planning to introduce DMPA-SC or other new contraceptive methods in settings where stakeholder expectations for measureable results for decision-making are high.

## Highlights

• From 2014 to 2016, the MOHs of Burkina Faso, Niger, Senegal and Uganda launched pilot introductions of the novel injectable contraceptive DMPA-SC.
• This manuscript describes a four-phase approach to monitoring DMPA-SC pilot introduction across the four countries.
• Existing national family planning registers were used to monitor DMPA-SC pilot introductions, with modifications.
• Comprehensive data from DMPA-SC pilot introduction informed decisions about future investments in the product and scaling product availability nationally.

## 1 Introduction

**Fig. 1:**
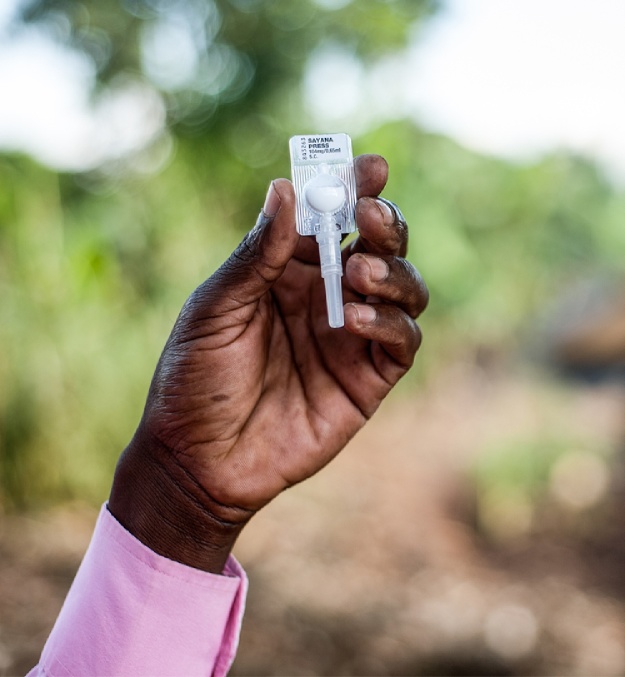
DMPA-SC unit. (Photo: PATH/Will Boase).

Contraceptive use in sub-Saharan Africa continues to be low, where only 26% of married or in-union women aged 15 to 49 are using some form of modern contraception (Population Reference Bureau, 2018). This region has the highest level of unmet need—24% of women of reproductive age wish to delay or stop childbearing, but do not use a modern method of contraception (United Nations, 2015). A new injectable contraceptive, subcutaneous depot medroxyprogesterone acetate (subcutaneous DMPA or DMPA-SC), offers potential to improve contraceptive access and uptake, especially in remote locations, by expanding the range of methods available to women outside of clinic settings (Fig. 1). DMPA-SC is easy to use and requires minimal training, making it especially suitable for administration by lay health workers in peripheral facilities, through community-based distribution (CBD), and even by women themselves through self-injection. Evidence suggests that adding a new contraceptive method to the mix or expanding geographic access to existing methods attracts new contraceptive users and increases contraceptive prevalence (Jain, 1989; Ross & Stover, 2013).

DMPA-SC is a three-month, progestin-only injectable contraceptive administered below the skin into the fat. The most widely available DMPA-SC product, Sayana® Press, is manufactured by Pfizer Inc. A lower-dose formulation and presentation of the intramuscular contraceptive Depo-Provera®, Sayana Press contains 104 mg per 0.65 mL dose of DMPA and combines the drug and needle in the prefilled BD Uniject^TM^ injection system. (Sayana Press and Depo-Provera are registered trademarks of Pfizer Inc. and Uniject is a trademark of BD.)

At the 2012 London Summit on family planning, more than 70 governments and organizations made unprecedented political and financial commitments to support the right of women and girls to decide—freely and autonomously—whether, when, and how many children they have. The governments of Burkina Faso, Niger, Senegal, and Uganda—among others—set ambitious objectives to reach additional users of modern contraception and increase contraceptive prevalence rates (CPR) by 2020. To help reach their objectives, these countries made specific commitments related to scaling up community-based distribution—including CBD of injectables—and to support innovation in family planning service delivery by introducing DMPA-SC into their national family planning programs (Family Planning, 2018).

Following these commitments, PATH and key partners collaborated with ministries of health (MOHs) in Burkina Faso, Niger, Senegal, and Uganda to launch the first pilot introductions of DMPA-SC in sub-Saharan Africa. The specific DMPA-SC product introduced and monitored in these four countries was Sayana^®^ Press. This method was introduced through existing family planning delivery channels in the public, nongovernmental organization (NGO), and commercial sectors in both urban and rural areas from July 2014 through June 2016. In each country, the method was also offered in new delivery channels to increase access outside of clinics, such as through outreach from peripheral facilities, and provision by community health workers (CHWs) at health huts, or through CBD. The MOHs of Burkina Faso and Senegal elected to offer DMPA-SC at all levels of the health system in the countries’ four most populous regions. Uganda’s MOH introduced DMPA-SC in 28 districts through CBD by trained CHWs. The MOH of Niger introduced the product via CHWs in remote districts—at peripheral health huts in two districts and through private NGO-sector CBD agents in two additional districts (four districts total) (Stout et al., 2018).

Global and national stakeholders had key questions about these pilot introductions to inform future investments in the product and decisions about scaling up product availability and service-delivery innovations nationally. These questions included the number of DMPA-SC doses that would be administered; the extent to which DMPA-SC would appeal to first-time users of modern contraception, as well as adolescent girls and young women; whether DMPA-SC would add value to family planning programs or simply replace intramuscular DMPA (DMPA-IM) or other modern methods; and the effect of introducing an injectable at the community level for the first time. To answer stakeholders’ key questions, assess the reach of these pilot introductions, and inform mid-project course corrections, PATH and partners decided to generate evidence through the collection of monitoring data across the four pilot countries.

One method for assessing the pilot introductions would be to use data from existing country monitoring systems—national health information systems (HIS)—which send family planning service statistics to the central level. However, these systems often are not a reliable source for project-specific data for several reasons. First, there is generally a significant delay in the availability of data produced by these systems (Bertrand, Magnani, & Rutenberg, 1996), with service statistics reaching the central level only twice a year or even less often. National HIS also are not able to capture specific data for new methods introduced on a pilot basis. Rather than collect data on individual products (e.g., DMPA-SC, DMPA-IM), these systems often aggregate data across method types (e.g., pills, injectables, implants). Finally, national HIS are not able to disaggregate data for new delivery channels under a pilot project (e.g., CBD vs facility-level data), instead aggregating data from across service-delivery channels. Thus, relying on national HIS cannot provide disaggregated data specific to the introduction of a new method or from new delivery channels, such as CBD.

A range of tools and resources exist for monitoring and evaluation (M&E) of family planning programs (Adamou et al., 2013; Barden O’Fallon & Bisgrove, 2016; MEASURE Evaluation, 2018). Research studies evaluating pilot introductions of new contraceptive methods—including review of service statistics in some cases—are also well documented in the literature (Garza-Flores et al., 1998; Gribble, Lundgren, Velasquez, & Anastasi, 2008; Hubacher, Akora, Masaba, Chen, & Veena, 2014; Lundgren et al., 2012; Zenger, Shuhua, & Huimin, 1995). However, there is a lack of published material to guide the specific process for using monitoring systems to generate data on pilot introductions of new contraceptive technologies in national family planning programs. An assessment of family planning M&E strengths, weaknesses, and gaps, published by MEASURE Evaluation highlighted the need for mechanisms for timely and rapid data collection in this field and recommended making better use of existing in-country data, service statistics, and HIS (Barden O’Fallon & Bisgrove, 2016). The authors further called attention to the need to balance the burden of data collection experienced by local program personnel with the needs of in-country HIS, donors, and other stakeholders (Barden O’Fallon & Bisgrove, 2016).

In view of the inability to use country HIS to collect project-specific data and the limited guidance for using monitoring systems to evaluate pilot projects, we consulted with local and global stakeholders to design and implement a multicountry (“global”) monitoring system across four countries. While each country opted for a different product introduction strategy, all agreed to measure a common set of indicators for the pilot project in order to produce timely, disaggregated data for decision-making and allow analysis of data in relation to different training and introduction strategies. In order to collect the data we needed, we made minimal revisions to existing family planning registers and developed project-specific reporting forms to disaggregate DMPA-SC from other injectables. Facility supervisors and/or district personnel completed our project-specific data summary reporting forms and also continued to report aggregated data on injectables using HIS forms per standard procedures, ensuring data on the new method would also be included in the countries’ aggregated service statistics. Depending on the country, we picked up the disaggregated data from the health facility or district during routine supervision and entered them into a project database. This approach resulted in two distinct data reporting flows: one that was project-specific and disaggregated DMPA-SC, and another that aggregated DMPA-SC with other injectables for routine HIS reporting. While creating parallel monitoring systems is resource intensive and not sustainable long-term, PATH and partners leveraged existing family planning registers and private-sector data collection systems to the extent possible to capture data specific to the new method and delivery channels while minimizing the burden of additional data collection on health workers.

This manuscript describes how the monitoring system for pilot introduction of DMPA-SC was designed and implemented, with an emphasis on sharing insights for program planners and implementers who are considering introduction of DMPA-SC or other new contraceptive methods.

## 2 Design and outcomes: a four-phase approach to monitoring pilot introduction of DMPA-SC

**Fig. 2:**
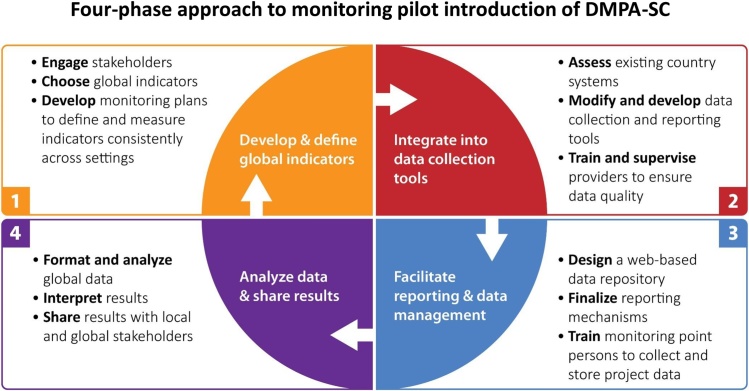
Four-phase approach to monitoring pilot introduction of DMPA-SC.

PATH implemented a four-phase process in 2014 and 2015 to develop a system for monitoring DMPA-SC pilot introduction in Burkina Faso, Niger, Senegal, and Uganda—consulting and collaborating with MOHs and country implementing partners at every step (Fig. 2). The four phases were:

• Develop and define global indicators.
• Integrate global indicators into country data collection tools.
• Facilitate consistent reporting and data management.
• Analyze and interpret data and share results.

While the term “global indicators” refers to cross-cutting indicators for the four project countries, they are also global in the sense that they may be applied to new settings introducing DMPA-SC.

### 2.1 Phase 1: develop and define global indicators

#### 2.1.1 Choosing appropriate indicators

We first consulted family planning indicator compendiums and drafted a set of suggested process and outcome indicators to track progress of provider training and DMPA-SC introduction, and to generate data for making decisions during pilot introduction. We then engaged donors and country partners—including MOHs and NGO implementing partners—in a process of reviewing and refining the global indicators to provide a final set that all stakeholders agreed to collect across countries, if relevant to their country introduction strategy. This process involved technical visits to pilot introduction countries in which we reviewed and debated suggested indicators with family planning leaders from MOHs and relevant personnel from implementing partner organizations. For each indicator, these stakeholders considered the importance and usefulness, relevance to their country introduction strategy, and feasibility of data collection. Country technical visits also included field missions to health facilities and district health offices to further assess feasibility of data collection and reporting, and to prepare for Phase 2 (described below). Table 1 lists the global pilot project indicators and the purpose of each indicator. More detailed information about the global indicator definitions, data requirements, recommended reporting periods, and measurement levels is available in the online resource “Global monitoring guide for the introduction of subcutaneous DMPA (DMPA-SC)” (PATH, 2018).

**Table 1:** Global monitoring indicators for pilot introduction of DMPA-SC.

Global monitoring indicator	Purpose
Number of providers trained, by type	• This process indicator documents the number and type(s) of family planning service providers trained in the provision of DMPA-SC; helps track whether training is progressing as expected.
Number of DMPA-SC doses administered to clients	• This outcome indicator documents the number of DMPA-SC doses administered to clients, independent from other injectable products. • Provides the denominator for indicators on new users, switching from DMPA-IM, and switching from other modern methods.
Number and percent of DMPA-SC doses administered to first-time users of modern contraception (“new users”)^a^	• This outcome indicator documents the total number of new users of modern contraception reached with DMPA-SC, and the share of total DMPA-SC doses administered to first-time users, by health system level where relevant. • Helps determine the extent to which the product is reaching new users, as opposed to users who had previously used another modern method. • The denominator for the percent indicator is the number of DMPA-SC doses administered to clients.
Number and percent of DMPA-SC doses administered to clients under age 20, ages 20 to 24, ages 25 and older (Niger, Senegal, and Uganda only)	• This outcome indicator documents the extent to which providers administer DMPA-SC doses to adolescent girls and young women. • May indicate whether DMPA-SC is an attractive method choice for adolescent girls and young women. • May highlight areas where additional training on provision of family planning methods (and/or injectables) to adolescents could be needed. • The denominator for the percent indicator is the sum of doses administered to clients in each age category.
Number and percent of DMPA-SC doses administered to clients who switched from DMPA-IM (Burkina Faso, Senegal, and Uganda only)	• This outcome indicator documents the number and proportion of DMPA-SC doses administered to clients switching from DMPA-IM, in order to track an early concern of stakeholders that DMPA-SC—a more expensive product at the time—would potentially replace DMPA-IM. • May indicate whether women and/or providers prefer DMPA-SC to DMPA-IM. • May indicate need to follow up with providers during supervision to ensure DMPA-SC is not promoted as a replacement for DMPA-IM. • The denominator for the percent indicator is the number of DMPA-SC doses administered to clients.
Number and percent of DMPA-SC doses administered to clients who switched from modern methods other than DMPA-IM (Burkina Faso and Senegal only)	• This outcome indicator documents the number of DMPA-SC doses administered to clients switching from modern methods other than DMPA-IM. • The denominator for the percent indicator is the number of DMPA-SC doses administered to clients.
Number of DMPA-IM doses administered to clients	• This outcome indicator documents the volume of DMPA-IM doses administered to clients, independent from other injectable products. • Provides input for the numerator and denominator for the indicator on relative proportions of DMPA-SC and DMPA-IM administered, by level.
Relative proportions of DMPA-SC and DMPA-IM administered, by level (where both methods are available)	• This outcome indicator documents the relative share of the market comprised of DMPA-SC and of DMPA-IM, by level, where providers offer both methods. • May indicate the preference of women and/or providers for each method, though factors such as provider skill level and potential bias should also be considered. • Numerators include the number of doses of DMPA-SC and DMPA-IM administered to clients. The denominator is the sum of the number of doses of DMPA-SC and DMPA-IM administered to clients.
Number of DMPA-SC doses distributed to health facilities	• This process indicator monitors functioning of the commodities distribution system. Documents the extent to which DMPA-SC is delivered and available for clients.
Number and percent of facilities with a stockout of DMPA-SC	• This outcome indicator documents the extent of DMPA-SC stockouts and contextualizes trends in DMPA-SC consumption and in the overall method mix. • Helps identify locations where the distribution system and/or facility stock management practices may require reinforcement. • The denominator for the percent indicator is the number of facilities active in the provision of DMPA-SC that reported during the same period.
Number of facilities active in the provision of DMPA-SC that reported this period	• This process indicator documents the number of facilities that reported on DMPA-SC in a given period. • Provides input on data completeness. • Provides the denominator for the percent of facilities with a stockout of DMPA-SC.

a A first-time user of modern contraception—also referred to as “new user”—is defined as a client who has elected to use modern contraception for the first time in her life.

The list of global indicators was intentionally limited to a small group of essential indicators related to stakeholder’s specific questions. Keeping the number of indicators manageable for health workers who must balance patient care with data collection increases the likelihood of receiving complete and accurate data.

#### 2.1.2 Defining and measuring indicators consistently

In practice, not all family planning programs define indicators in exactly the same way. For this project, it was essential to define indicators consistently both *within* and *across* countries to ensure any data we collected would be accurate and comparable across settings. During this phase, for example, we encountered the following definitions of “new user”: a client who was using a particular method for the first time; a client who had not used a method in the previous 12 months and was returning to contraception; a client who was adopting any modern method of contraception for the first time. Unifying indicator definitions across settings was a key challenge encountered during the monitoring system design and reaching consensus among all stakeholders was time consuming. To ensure consistent definitions and comparable data across countries, this project implemented standard definitions and measurement of all indicators through detailed monitoring plans that clearly defined each indicator as well as the data requirements and desired disaggregations. We trained providers on the correct application of indicator definitions before the start of data collection, and once data collection began we continued to reinforce these definitions through routine supervision. We defined a new user as someone electing to use modern contraception for the very first time in her life (e.g. has never previously used any modern method).

#### 2.1.3 Tailoring indicators to country contexts

Some indicators—such as DMPA-SC doses administered by client age group and switching from DMPA-IM or from other modern methods—were not collected in every country. For example, stakeholders in Niger elected not to collect data on client switching from DMPA-IM because it would theoretically be low to nonexistent, since DMPA-SC was introduced only at the community level, where DMPA-IM was not available. The indicator on age of clients was optional, and Burkina Faso elected not to report this in order to reduce the burden of additional data reporting on facility supervisors. Countries also developed their own sets of indicators to track pilot introduction, which were lengthier and reflected country-specific data needs or interests. For example, stakeholders in Uganda chose to track the number of visits to community health workers at which women were referred to a health center and the reason for referral (e.g., for a long-term or permanent method, side effects management, or injection site reaction). While data for these indicators were used to answer country-specific questions about introduction, they were not included in routine global analysis and reporting for the project.

### 2.2 Phase 2: integrate global indicators into country data collection tools

In the second phase, PATH worked with country partners to integrate the global—and in some cases, country-specific—indicators into existing service delivery data collection tools, and to develop new registers and reporting tools where necessary.

#### 2.2.1 Assessing country systems

Through a series of visits to service delivery points (SDPs) and district health offices, we first assessed existing country data collection tools and HIS reporting systems. The goal of this activity was simply to understand how monitoring data usually flow from the points of product distribution and family planning service delivery up to the central level under the existing system. This included some assessment of the completeness and accuracy of current data collection and reporting at different SDPs. In each country, we reviewed stock management procedures and records, individual client record forms, family planning registers, and routine facility and district-level reports in order to understand what data were being collected, disaggregation levels, and frequency of reporting to the HIS. This process allowed us to identify gaps where the current system would be unable to capture data required for the pilot project. For example, we learned that existing HIS reporting forms would not allow for DMPA-SC to be disaggregated from other injectables or for doses administered through CBD to be captured separately from doses administered at health facilities. This step was critical for understanding that the project would need to modify or introduce new tools in order to collect DMPA-SC disaggregated data, and develop a plan for picking up the data at the facility or district level, since they would not be reported to the HIS. Since our project was introducing DMPA-SC through a pilot, it would be neither feasible nor appropriate to revise HIS systems to collect the disaggregated data we desired.

#### 2.2.2 Modifying data collection procedures with existing tools

Once we identified the differences between the existing data collection system and the system desired under pilot introduction, PATH worked with country partners to modify data collection procedures for existing tools to capture data required for the global indicators. For example, to ensure DMPA-SC would be differentiated from other injectables in family planning registers, stakeholders in certain countries decided that health workers would simply write in the name of the method (i.e., Sayana Press or DMPA-IM) rather than “injectable.” In other settings with an “injectables” column in the register, a line was drawn down the middle to create two separate columns—one for DMPA-SC and one for DMPA-IM. These approaches allowed providers to continue using existing family planning registers with only slight modifications to how they collected data on injectables. Other revisions included making sure that the definition of a “new user” was consistently defined as a client who elected to use modern contraception for the very first time, and that there was a way to capture switching to DMPA-SC from other methods. The MOH in Burkina Faso was preparing to reprint the family planning registers, and agreed to make certain revisions—such as the definition of “new user”—directly in the new registers. In other cases, new instructions for completing registers and forms were developed and distributed in order to accommodate the pilot project data requirements without having to reprint registers, with any updates to data collection and reporting procedures reinforced during training and supervision.

#### 2.2.3 Creating and introducing new tools

**Fig. 3:**
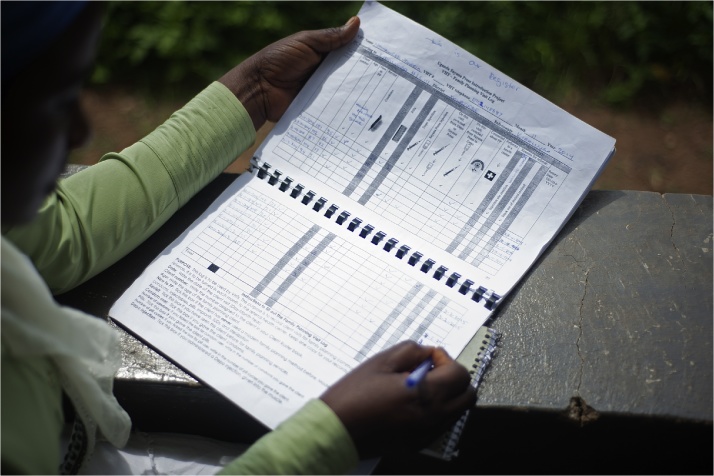
A community health worker in Uganda fills out the new family planning visit log designed to collect CBD data under the pilot project and meet local data requirements. (Photo: PATH/Will Boase).

In some cases, we designed and introduced new data collection and reporting tools to capture global indicator data and meet local data requirements. For example, in Uganda, PATH worked with the MOH to extend the monitoring system to the community level for the pilot project. Previously, provision of family planning services at the community level was minimal and primarily driven by NGO implementing partners, so there were no national registers for CHW service delivery. Any data CHWs did collect were aggregated with referral facility data for HIS reporting, making it impossible to calculate the contribution of CHWs to the family planning program. We reviewed all the existing data collection tools from various partners and the HIS data requirements and developed a new CHW family planning visit log to collect pilot-specific data and meet the data needs of different stakeholders (Fig. 3). Project-specific data reporting forms were also developed in each country to facilitate reporting summary data for DMPA-SC, and disaggregating by service delivery channel, to the project database described in phase 3. Facility supervisors and/or district personnel (depending on the country) would complete the project’s summary reporting tools in addition to the usual HIS forms.

In Senegal, the Informed Push Model (IPM)—implemented to reduce stockouts to below 2% in Senegal’s public sector—was able to provide data on DMPA-SC doses consumed, a proxy for total doses administered. According to IPM’s Project Director, doses consumed were calculated by tracking the change between the amount of stock at SDPs at the end of the previous delivery and the amount of usable stock remaining upon arrival at the current delivery (L. Hasselback, MPA, MA, personal communication, September 2014). However, the IPM did not collect detailed service statistics, so PATH implemented a sentinel site system—collection of data from a sample of 35 SDPs—to retrieve data on DMPA-SC doses administered to new users, women of different age groups, and those switching from DMPA-IM or other modern methods. In coordination with the MOH, sentinel sites were selected from each of the four pilot regions on the basis that they had frequent family planning clients—notably those using injectables—and were accessible during all periods of the year. Project monitoring staff visited the sites at least quarterly to collect the necessary data from the family planning registers. Together with data on doses distributed, doses consumed (proxy for doses administered), and stockouts from IPM, the sentinel site data fulfilled all of the data requirements for global monitoring during the pilot period. While results from the sentinel sites provided valuable insights into pilot introduction in Senegal, they cannot be generalized to the entire introduction initiative due to purposive sampling.

#### 2.2.4 Training providers for data collection and reporting

Phase 2 also involved training providers in data collection, and facility supervisors in compiling summary data in project-specific reporting forms. In order to streamline the training process, we decided to train service providers and facility supervisors on data collection and reporting at the same time they received training on DMPA-SC service provision. We integrated a module on data collection and reporting into the broader training curricula on DMPA-SC service provision, so that these personnel would be ready to collect data as soon as they began administering the new product. Project staff responsible for coordinating monitoring efforts in each of the countries (referred to as monitoring point persons) also carried out district visits to orient district data managers to the project and quarterly reporting templates, where relevant.

### 2.3 Phase 3: facilitate consistent reporting and data management

#### 2.3.1 Developing data flow diagrams

After developing a plan to integrate data for the global indicators into existing data collection tools and developing new procedures and forms where needed, PATH worked with country partners to ensure that all necessary data would be captured and consistently reported in the project’s global monitoring system. We developed a unique data flow diagram for each country to illustrate data collection and movement through the monitoring system from points of product distribution and client visits to reporting directly to PATH—the organization responsible for coordinating the project’s global monitoring system—or to the HIS. Data flow diagrams are like a visual monitoring plan, providing detail on what data are collected, when they are collected (e.g., during client visits), tool(s) used, person collecting data, and frequency of collection. They also illustrate the reporting hierarchy—for example, the points at which data enter the national HIS or are collected by project monitoring focal points to be entered into the global monitoring database. Project data flow diagrams can be revisited periodically to determine whether the monitoring plan is functioning as expected, to identify obstacles, and to guide adjustments to optimize the data collection system.

#### 2.3.2 Finalizing reporting mechanisms

**Fig. 4:**
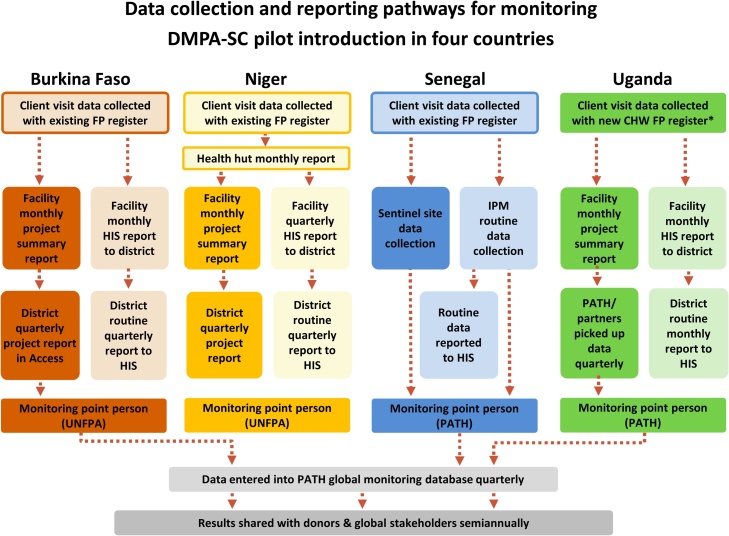
Data collection and reporting pathways. In Uganda, we designed and implemented a new register for CHWs to collect data on community-based delivery of family planning. In Burkina Faso, Niger, and Senegal, we used existing national family planning registers and simply modified how providers filled out the forms in order to capture disaggregated data for the project. Abbreviations: FP, family planning; CHW, community health worker; HIS, health information system; IPM, Informed Push Model, UNFPA, United Nations Population Fund.

During this phase, we worked with country partners to ensure that data from the pilot project would be reported both to the project’s global monitoring system and the national HIS for inclusion in the country’s family planning service statistics. In all countries, data on the global indicators were collected by providers and aggregated first at the facility-level using new project-specific forms or reporting procedures. In Senegal and Uganda, project monitoring point persons generally picked up these data directly from facilities. In Burkina Faso and Niger, new reporting forms capturing pilot-specific data were also implemented at the district level, where project monitoring point persons in each country would retrieve data and report to PATH on a quarterly basis. Paper forms were used throughout the pilot introduction countries, with the exception of district reporting in Burkina Faso, which was facilitated via a Microsoft Access database, allowing electronic transmission of project data to the monitoring point person before entry into the project’s global database. Standard HIS reporting procedures were carried out as usual to ensure the inclusion of DMPA-SC data (aggregated into an “injectables” category) in national family planning service statistics. Fig. 4 depicts the flow of data through the project’s monitoring system in each country and as well as reporting to the HIS. Data from NGO implementing partners were also integrated into reporting processes, either at the district level or through separate reports sent to PATH.

#### 2.3.3 Collecting and storing project data

To facilitate country reporting and global data storage, PATH developed a customized web-based database using Microsoft SharePoint. The electronic data entry interface for each country was identical to the reporting forms used in each setting to promote ease of data entry. During in-country visits and through remote technical assistance, the project’s global M&E officer trained monitoring point persons in each country to enter pilot introduction data into the database. This platform allowed data to be entered on a rolling basis or at the close of each quarter, and these data were immediately available to the project’s global M&E officer. Project managers in each country also had access to the data via the SharePoint database to guide local monitoring and supportive supervision.

This system streamlined data management by eliminating the potential for disparate reporting formats and version control issues that can result from emailing reports and datasets. It also simplified the process for aggregating data across all four pilot countries, as data were easily exported to Excel for management and formatting, facilitating individual country and comparative analysis. This approach helped meet the data needs of global and national stakeholders who required specific and timely data for making decisions on DMPA-SC introduction and scale, as it was not feasible for national HIS to integrate data collection for a product introduced through limited-scale pilot introductions.

### 2.4 Phase 4: analyze and interpret data and share results

**Fig. 5:**
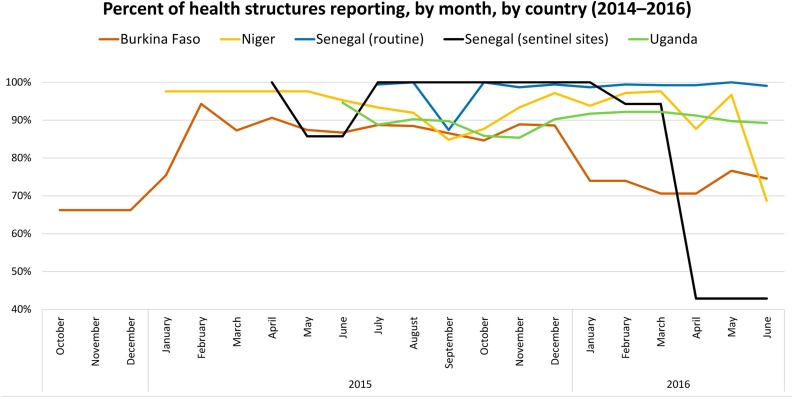
Percent of health structures reporting, by month, by country (2014–2016).

Each quarter, PATH cleaned data and worked with country partners to correct errors and fill gaps. Our process resulted in a high-level of data completeness across each of the four countries. Fig. 5 depicts the percentage of health structures reporting over time in each country, from the conclusion of provider training through the end of the pilot. In each country, with the exception of Uganda, health structures represent SDPs (e.g., health facilities, health huts) that offered DMPA-SC. In Uganda, DMPA-SC was only available through CBD, so health facilities aggregated and reported data each month from the CHWs they supervised. Sentinel site reporting in Senegal dropped off significantly during the last quarter of introduction, as the anchor project of the partner responsible for supervising and reporting data from 20 of the sentinel sites had closed out.

Twice a year, the global dataset was analyzed to measure cumulative progress and depict trends. PATH used Power Query (an Excel application) to consolidate the country datasets and format the data, and used Tableau and Excel for data analysis and visualizations. Results were first shared back with country project teams to elicit their feedback on results and trends and assist with data interpretation. This step was essential in order to provide global stakeholders with the important contextual information needed to interpret the results correctly. For example, it was important to highlight that frequent stockouts of DMPA-SC in two countries were precipitated by a large quantity of product expiring, and to share the steps each country took to ameliorate stockouts and prevent them from recurring. We formally shared results semiannually with global and country stakeholders to inform decision-making, and disseminated high-level results to a broad stakeholder audience.

The final project results are reported by Stout et al. (2018). Below, we describe several lessons learned from our experience implementing a global monitoring system to track DMPA-SC pilot introduction across four countries.

## 3 Lessons learned

Producing high-quality data for decision-making relies on strong monitoring system design and robust data collection systems. In the four countries where we implemented pilot introduction of DMPA-SC, national HIS did not produce timely, method-specific data that could be used for prompt decision-making. While creating parallel data collection systems is project dependent and unsustainable long term, here it was necessary to obtain results to inform national governments’ decisions about national scale. Lessons learned from our experience monitoring pilot introduction of DMPA-SC provide insights that may benefit country governments and implementing partners planning similar introductions of DMPA-SC or other novel contraceptive methods.

### 3.1 Start early

Choosing project indicators and designing the data collection and management systems should coincide with planning the overall project strategy. Several iterations of tools may need to be tested before data collection systems are finalized. Delays in implementing the monitoring system will translate to delays in data collection and reporting, which could result in missing important data from the early phases of introduction.

### 3.2 Finalize indicators and develop detailed monitoring plans

To produce meaningful results, stakeholders within and across countries—including providers who collect the data—must share common definitions of indicators and interpret them consistently (e.g., the definition of a “new user”). It is also important to ensure data are measured and reported uniformly across country settings to account for disaggregation by region/district, public- or private NGO-sector, facility- vs community-level delivery, and reporting period. If national programs, NGOs, or service providers within countries measure indicators differently, the data will not be comparable. Developing a detailed monitoring plan that clearly defines each indicator as well as the desired disaggregations, calculations, data sources, and reporting frequency will allow analysis of global indicators across country settings, sectors, and levels of the health system, and assist in drawing conclusions about results achieved through various introduction strategies.

### 3.3 Collaborate with local stakeholders to build an appropriate monitoring system

Working closely with ministries of health and other national stakeholders in planning and implementation was critical to establishing an appropriate monitoring system that effectively leveraged existing tools and systems. Keeping global indicators to a minimum and building data collection into existing family planning registers and reporting timelines to the extent possible minimizes the burden on health systems and maximizes opportunity for sustainability.

### 3.4 Conduct site visits

Site visits are indispensable during the planning phase to understand how data are currently collected, which tools are used, what challenges health workers encounter, how data flow through the existing monitoring system, and where bottlenecks occur. While statisticians at the central level can provide data collection forms and describe the national monitoring system, there may be discrepancies between how the system works in theory and how it is implemented in the field. For example, at peripheral levels of the health system where supervision may be weak, data collection forms may be out of date or used incorrectly, resulting in inaccurate or incomplete reporting. Site visits during the implementation phase, in the form of ongoing supervision, are essential to make sure data are being collected and reported accurately.

### 3.5 Disaggregate data in new delivery channels

In settings where DMPA-SC was introduced through CBD (Uganda), as the first-ever offer of an injectable contraceptive by mobile outreach workers (Burkina Faso) or by CHWs in rural health huts (Niger), data on the number of doses administered via each delivery channel were needed to analyze the effect of this service innovation. If CBD data are rolled into those of the referral facility, it will not be possible to measure the new component of a program. In Burkina Faso, inaccurate application of indicator definitions by outreach workers resulted in the project being unable to disaggregate mobile outreach data and discern the contribution of outreach services to new users and overall product uptake. This challenge underscores the importance of reinforcing consistent indicator definitions and measurement at multiple levels—e.g., through monitoring plans, provider and supervisor training, and ongoing routine supervision.

### 3.6 Pilot test data collection and reporting tools and strategies

Introducing or modifying data collection and reporting tools requires piloting the tools to test providers’ comprehension, appropriateness to context, and effectiveness of reporting mechanisms. Several iterations may be needed before tools are ready for implementation. During the initial phase of developing the tools, it is vital to ensure smooth flow of data into existing national data systems—for example, by testing whether data from new forms easily transfer to existing monthly or quarterly reporting HIS templates—as well as the project’s global monitoring system. Developing the tools four to six weeks prior to provider training will allow adequate time to pilot test, revise, and print the tools before rolling the product out to SDPs.

### 3.7 Train providers on the monitoring tools

Training health workers and facility supervisors on data collection and reporting at the same time as training on the new contraceptive method is efficient and timely. Participants should have the opportunity to practice collecting data with new or existing forms by using case studies or working in pairs. Training providers on data collection after service-delivery training—e.g., during routine supervision visits—is costlier and requires correcting established habits. Ongoing supervision will still be needed to reinforce consistent and correct application of indicator definitions, proper data collection, and good reporting practices.

### 3.8 Conduct routine data quality checks

It is essential to conduct routine data quality checks early and often. Checking data quality at multiple levels—such as when facility supervisors aggregate data, project field staff conduct supervision, and during data cleaning by the project’s global M&E officer—provide various opportunities to identify and correct data errors, and reinforce correct data collection and reporting procedures. Early on in the project, routine data cleaning revealed high numbers of doses administered to clients switching from DMPA-IM in multiple countries. We investigated the high levels of switching and discovered a misconception among some providers that DMPA-SC was intended to replace DMPA-IM. Project staff then corrected this misunderstanding through targeted supervision visits.

## 4 Conclusion

Our four-phase approach to monitoring DMPA-SC pilot introduction resulted in high-quality, comparable data across four countries. We encountered key challenges during monitoring system design and implementation, including unifying indicator definitions across countries, providers in some settings initially misunderstanding that DMPA-SC was intended to replace DMPA-IM, and mobile outreach workers in Burkina Faso incorrectly applying indicator definitions resulting in unusable outreach data. Using project results to guide decision-making, governments in all four countries made the decision to scale up DMPA-SC nationwide, and expansion is currently underway. To support national scale-up efforts after the pilot introductions concluded, PATH participated in the process to revise national HIS to include relevant project- and/or method-specific indicators, as appropriate. MOHs in some countries decided to incorporate the disaggregation of injectables by type, methods administered by client age group, or the updated new user definition into national HIS going forward. Strong monitoring system design is essential to assess a project’s performance and to measure project results. The monitoring system we designed met a key project objective to make timely, comprehensive data available to global and national stakeholders to inform project management, future investments in the product, and decisions about national scale.

## Conflicts of interest

None.

## Role of the funding source

This work was supported by the Bill & Melinda Gates Foundation, Seattle, WA [award number OPP1060986]; and the Children’s Investment Fund Foundation, London, United Kingdom [Request #333]. The funding sources did not play a role in study design; the collection, analysis, and interpretation of data; the writing of the report; or the decision to submit the article for publication.
