# Supplementary material for: Designing a global monitoring system for pilot introduction of a new contraceptive technology, subcutaneous DMPA (DMPA-SC)
Source: Eval Program Plann. 2018 Jun;68:185–93. doi: 10.1016/j.evalprogplan.2018.03.004 (PMC5958241; doi:10.1016/j.evalprogplan.2018.03.004)
Supplement: Supplementary file 1 [file mmc1.pdf]

PATH

Global Monitoring Guide for the Introduction of Subcutaneous DMPA (DMPA-SC)

In collaboration with ministries of health and key partners, PATH coordinated pilot introduction of the injectable contraceptive subcutaneous depot medroxyprogesterone acetate (subcutaneous DMPA or DMPA-SC) in Burkina Faso, Niger, Senegal, and Uganda from July 2014 through June 2016. DMPA-SC is a three-month, progestin-only injectable contraceptive. Sayana® Press[1], the most widely available DMPA-SC product, is manufactured by Pfizer Inc. and combines the drug and needle in the prefilled BD Uniject™[2] injection device. PATH worked in partnership with local and global stakeholders to build consensus on a set of global indicators for monitoring DMPA-SC pilot introduction across the four countries. Indicators were selected based on key interests of national stakeholders and donors to inform decisions regarding scale-up and future investments in DMPA-SC.

In addition to tracking volumes of doses administered, PATH's approach to monitoring DMPA-SC pilot introduction measured indicators related to first-time users ("new users") of modern contraception, adolescent girls and young women, and switching to DMPA-SC from other modern methods, especially intramuscular DMPA (DMPA-IM). During the design of the monitoring system, PATH paid careful attention to defining indicators consistently within each country, as well as globally. Harmonizing indicator definitions across countries was essential to allow for cross-country analysis of monitoring data, which provided rich information on the outcomes of different training and introduction approaches. This guide summarizes the global indicators for pilot introduction, their definitions, and suggested data sources and measurement levels. These can be modified for various country settings or program needs.

No.	Indicator	Definition and data requirements	Purpose	Periodicity, reporting, and measurement level	Data source(s)
**Training**		***Select programmatic considerations in italics***			
1.1	Number of provider trainings held on provision of DMPA-SC	The number of training events held on administration of DMPA-SC (and other family planning methods, as relevant)	• Documents the number of trainings held; can help ensure training is progressing as expected	• Collected on a rolling basis as training is implemented (monthly)<br>• Reported quarterly<br>• Disaggregated by district, sector, and facility level or provider type (depending on country context)	• Training records of MOH or NGO partner(s) responsible for provider training

[1] Sayana Press is a registered trademark of Pfizer Inc.
[2] Uniject is a trademark of BD.

1.2	Number of providers trained on the provision of DMPA-SC	The number of health workers who attended a full training (including theory and practical sessions) on administration of DMPA-SC (and other family planning methods, as relevant)	• Documents the number of health workers trained in the provision of DMPA-SC; can help ensure training is progressing as expected<br>• Denominator for indicator 1.4	• Collected on a rolling basis as training is implemented (monthly)<br>• Reported quarterly<br>• Disaggregated by district, sector, and facility level or provider type (depending on country context)	• Training records of MOH or NGO partner(s) responsible for provider training
1.3	Number of trained providers who attained competency in injectable delivery (DMPA-SC and/or DMPA-IM)	The number of providers trained who successfully completed theory and practicum and are certified to provide DMPA-SC (and/or DMPA-IM)<br><br>***Note:*** Competency standards, including both theory and injection practice, are country-specific and depend on the local context.	• Documents the number of health workers certified and potentially active in the provision of DMPA-SC<br>• Can help contextualize consumption data (e.g., higher volumes of DMPA-SC administered are typically seen where more health workers have been trained)<br>• Numerator for indicator 1.4	• Collected on a rolling basis as training is implemented (monthly)<br>• Reported quarterly<br>• Disaggregated by district, sector, and facility level or provider type (depending on country context)	• Provider observation checklists and post-training evaluation data<br>• Training records of MOH or NGO partner(s) responsible for provider training
1.4	Percentage of trained providers attaining competency in injectable delivery (DMPA-SC and/or DMPA-IM)	Out of the total number of providers trained, the percentage that were deemed competent in the provision of DMPA-SC<br><br>Numerator: 1.3<br>Denominator: 1.2	• Provides insight into the effectiveness of training and health workforce capacity to safely and effectively provide injectables	• Reported quarterly<br>• Disaggregated by district, sector, and facility level or provider type (depending on country context)	• Data for indicators 1.2 and 1.3
**Service delivery / consumption**					
2.1	Number of DMPA-SC doses administered to clients	The number of DMPA-SC doses injected into clients during the month<br><br>***Note:*** This indicator only represents the number of *doses* administered to women. It is not	• Determines consumption volumes of DMPA-SC<br>• *May help identify where additional provider training and support may be needed*<br>• *Consider behavior change communication and social*	• Collected monthly<br>• Reported quarterly<br>• Disaggregated by district, sector, and facility type or distribution channel (if relevant)	• Family planning client register

		equal to the number of DMPA-SC *users* at a point in time. Monitoring data are generally aggregated and thus unable to track individual use over time.	*marketing activities to help explain consumption patterns*<br>• Denominator for indicators 2.3, 2.7, and 2.9		
2.2	Number of DMPA-SC doses administered to new users	The number of DMPA-SC doses injected into new users during the month<br><br>***Note:*** A *new user* is defined as a woman who has elected to use a modern method of contraception for the very first time.<br><br>Clients can be counted as a new user only once, so the total number of doses administered to new users is equal to the number of new users.	• Indicates the total number of new users of modern contraception who opted to receive an injection of DMPA-SC<br>• Compared with data on new users who select other methods (if collected), will help determine which methods are preferred among new users<br>• Helps track progress toward global commitments (e.g., FP2020)<br>• Numerator for indicator 2.3	• Collected monthly<br>• Reported quarterly<br>• Disaggregated by district, sector, and facility type or distribution channel (if relevant)	• Family planning client register
2.3	Percentage of DMPA-SC doses administered to new users	The proportion of doses of DMPA-SC injected into first-time users of modern contraception<br><br>***Note:*** Modern methods consist of barrier, hormonal, and permanent methods such as male and female condoms, pills, injectables, implants, IUDs, diaphragms, spermicide foam/gel, male and female sterilization, lactational amenorrhea method, and emergency contraception.<br><br><u>Numerator</u>: 2.2<br><u>Denominator</u>: 2.1	• Documents the extent to which DMPA-SC doses are administered to first-time users of modern contraception<br>• *May indicate whether DMPA-SC is an attractive method choice for first-time users of modern contraception*<br>• ***Advanced monitoring:*** Programs that specifically track whether clients are new to family planning (vs. previously used any modern method) at *initial offer of DMPA-SC* may choose to use the total number of doses administered to clients initiating DMPA-SC as the denominator, eliminating reinjections from the equation.	• Reported quarterly<br>• Disaggregated by district, sector, and facility type or distribution channel (if relevant)	• Data for indicators 2.1 and 2.2

		Note: The percentage of doses administered to new users will decrease over time as more women return for reinjections, thus increasing the denominator (2.1). For this reason, it is also important to monitor the *number* of doses administered to new users. See next column for advanced monitoring tip.	Since a method can be initiated only once, this indicator would serve as a proxy for the percentage of DMPA-SC adopters new to family planning. This alternate indicator would be the percentage of clients initiating DMPA-SC that were new users of modern contraception.		
2.4	Number of DMPA-SC doses administered to women by age group:<br>a. Under age 20<br>b. Ages 20–24<br>c. Age 25 and older	The number of DMPA-SC doses injected in women under age 20, women ages 20–24, and women age 25 and older<br><br>***Note:*** Age category preferences may vary from country to country. These categories were selected due to alignment with the Demographic and Health Survey (DHS).	• Numerator for indicator 2.5	• Collected monthly<br>• Reported quarterly<br>• Disaggregated by district, sector, and facility type or distribution channel (if relevant)	• Family planning client register
2.5	Percentage of DMPA-SC doses administered to women:<br>a. Under age 20<br>b. Ages 20–24<br>c. Age 25 and older	The percentage of DMPA-SC doses injected in women under age 20, women ages 20–24, and women age 25 and older<br><br>For each age group:<br><u>Numerators</u>: 2.4a, 2.4b, and 2.4c<br><u>Denominator</u>: (2.4a + 2.4b + 2.4c)<br><br>***Note:*** The number of doses administered to women of each age range is divided by the sum of doses administered to women of all three age groups. This denominator is used rather than total doses administered, which	• Documents the extent to which DMPA-SC doses are administered to adolescents and young women<br>• *May indicate whether DMPA-SC is an attractive method choice for adolescents and younger women*<br>• *May highlight areas where additional training on provision of family planning methods (and/or injectables) to youth may be needed*<br>• ***Advanced monitoring:*** Programs that track *initial offer of DMPA-SC* (vs. previously used any modern method) may choose to only count age of clients at initial offer,	• Reported quarterly<br>• Disaggregated by district, sector, and facility type or distribution channel (if relevant)	• Data for indicators 2.4a, 2.4b, and 2.4c

		may not represent the true denominator since the ages of some women may not be documented in the family planning register.	which would more closely approximate the ages of DMPA-SC adopters. Counting age at every dose administered includes women every time they receive a reinjection.		
2.6	Number of DMPA-SC doses administered to women who switched from DMPA-IM (and/or other methods)	The number of DMPA-SC doses administered to women who were actively using DMPA-IM (or another method) and switched to DMPA-SC<br><br>***Note:*** Stakeholders in many country settings may be particularly interested in switching from DMPA-IM to DMPA-SC, though some may choose to track switching from other methods as well.	• Documents the number of DMPA-SC doses administered to clients switching from DMPA-IM (or the number of *times* clients switch from DMPA-IM to DMPA-SC)	• Collected monthly<br>• Reported quarterly<br>• Disaggregated by district, sector, and facility type or distribution channel (if relevant)	• Family planning client register
2.7	Percentage of DMPA-SC doses administered to women who switched from DMPA-IM (and/or other methods)	The percentage of DMPA-SC doses administered to women who were actively using DMPA-IM (or another method) and switched to DMPA-SC<br><br>Numerator: 2.6<br>Denominator: 2.1	• *May indicate whether women and/or health workers prefer DMPA-SC to DMPA-IM/other methods*<br>• *May indicate need to follow up with providers during supervision to ensure DMPA-SC is not promoted as a replacement for DMPA-IM*<br>• *May prompt investigation into DMPA-IM stock levels*<br>• ***Advanced monitoring:*** Programs that specifically track initial offer of DMPA-SC may choose to only count client method switching at initiation of the method (a proxy for counting *users* rather than *doses administered*) or choose to also count switching from DMPA-	• Reported quarterly<br>• Disaggregated by district, sector, and facility type or distribution channel (if relevant)	• Data for indicator 2.6

			SC back to DMPA-IM. Counting unidirectional switching with every dose administered does not account for the fact that women will switch back and forth between methods as stocks fluctuate. Indicator 2.7 may offer a better gauge of product preferences, where both methods are available.		
2.8	Number of DMPA-IM doses administered to clients	The number of DMPA-IM doses injected into clients during the month<br><br>***Note:*** This indicator only represents the number of *doses* administered to women. It is not equal to the number of DMPA-IM *users* at a point in time. Monitoring data are unable to track individual use over time.	• Determines DMPA-IM consumption volumes<br>• Provides information on the level and location of demand for DMPA-IM<br>• Input for numerator and denominator for indicator 2.9	• Collected monthly<br>• Reported quarterly<br>• Disaggregated by district, sector, and facility type or distribution channel (if relevant)	• Family planning client register
2.9	Relative proportions of DMPA-IM and DMPA-SC doses administered	The relative shares of DMPA-IM and DMPA-SC, where both methods are offered in parallel; the part that each method makes up of the whole (both methods combined)<br><br><u>Numerators</u>: 2.1 and 2.8<br><u>Denominator</u>: (2.1 + 2.8)<br><br>The number of DMPA-SC (or DMPA-IM) doses administered is divided by the sum of DMPA-SC and DMPA-IM doses administered to determine the proportion of each that make up the whole.	• Documents the share of the market comprised of DMPA-IM and comprised of DMPA-SC, where health workers are offering both methods<br>• May give an indication of preference for each method, though factors such as level of provider and provider bias should also be taken into consideration<br>• *Consider how stockouts of one or more methods may affect overall trends for this indicator.*	• Reported quarterly<br>• Disaggregated by district, sector, and facility type or distribution channel (if relevant)	• Data for indicators 2.1 and 2.8

		Note: The relative proportion of each method administered should add up to 1.0 or 100%.			
2.10	Number of facilities active in the provision of DMPA-SC that reported this period	The total number of health facilities having initiated offer of DMPA-SC that reported during the month	• Documents the number of facilities that reported on DMPA-SC in a given period<br>• Numerator for indicator 2.12<br>• Denominator for indicator 3.3	• Collected monthly<br>• Reported quarterly<br>• Disaggregated by district, sector, and facility type or distribution channel (if relevant)	• Facility or district reports
2.11	Total number of active facilities	The total number of facilities within the family planning program having initiated offer of DMPA-SC, regardless of current stockout status<br><br>***Note*:** A facility may become inactive if it loses trained health providers and its ability to provide contraceptive services.	• Denominator for indicator 2.12	***Note:*** Unless facilities are added or removed from the program (common early on in product introduction or scale-up), this indicator will remain constant.	• National family planning program or DMPA-SC introduction strategy documents
2.12	Percentage of facilities active in provision of DMPA-SC that reported this period	The percentage of health facilities offering DMPA-SC that reported during the period<br><br><u>Numerator</u>: 2.10<br><u>Denominator</u>: 2.11	• Tracks the extent to which facilities active in the offer of DMPA-SC are reporting service delivery data<br>• Will help inform how complete service delivery data are	• Reported quarterly<br>• Disaggregated by district, sector, and facility type or distribution channel (if relevant)	• Data from indicators 2.10 and 2.11
**Product distribution and stockouts**					
3.1	Number of doses of DMPA-SC distributed to health facilities	The number of DMPA-SC doses received into the stock of health facilities that are service delivery points for DMPA-SC, or that supply community health workers with product for community-based distribution	• Documents the extent to which DMPA-SC units are being delivered and are available for administration to clients<br>• Monitors the functioning of the family planning commodities distribution system	• Collected monthly<br>• Reported quarterly<br>• Disaggregated by district, sector, and facility type or distribution channel (if relevant)	• Records of distribution agency<br>• Health facility stock card
3.2	Number of active health facilities with a stockout of DMPA-SC	The number of health facilities offering DMPA-SC that did not have any usable doses of DMPA-SC on site (including in the	• *Identifies locations where the distribution system and/or facility stock management practices may require reinforcement*	• Collected monthly<br>• Reported quarterly<br>• Disaggregated by district, sector, and	• Records of distribution agency

		pharmacy and within the clinic) for one or more days during the month<br><br>***Note:*** A usable dose is a dose that is not damaged or expired.	• Numerator for indicator 3.3	facility type or distribution channel (if relevant)	• Health facility stock card
3.3	Percentage of active health facilities with a stockout of DMPA-SC	Out of the total number of facilities actively offering and reporting on DMPA-SC delivery, the percentage that did not have any usable doses of DMPA-SC on site (including in the pharmacy and within the clinic) for one or more days during the month<br><br>Numerator: 3.2<br>Denominator: 2.10<br><br>***Note:*** In many settings, stock data are collected and stored separately from client consumption data, so it is likely some facilities will not report consistently on stockouts. The number of facilities that reported is used as the denominator.	• Documents the extent to which the distribution system performs adequately<br>• Contextualizes trends in DMPA-SC consumption and in the overall method mix (i.e., if one method is stocked out over a period of time, it may explain increase in consumption of other methods)	• Collected monthly<br>• Reported quarterly<br>• Disaggregated by district, sector, and facility type or distribution channel (if relevant)	• Records of distribution agency<br>• Health facility stock card
